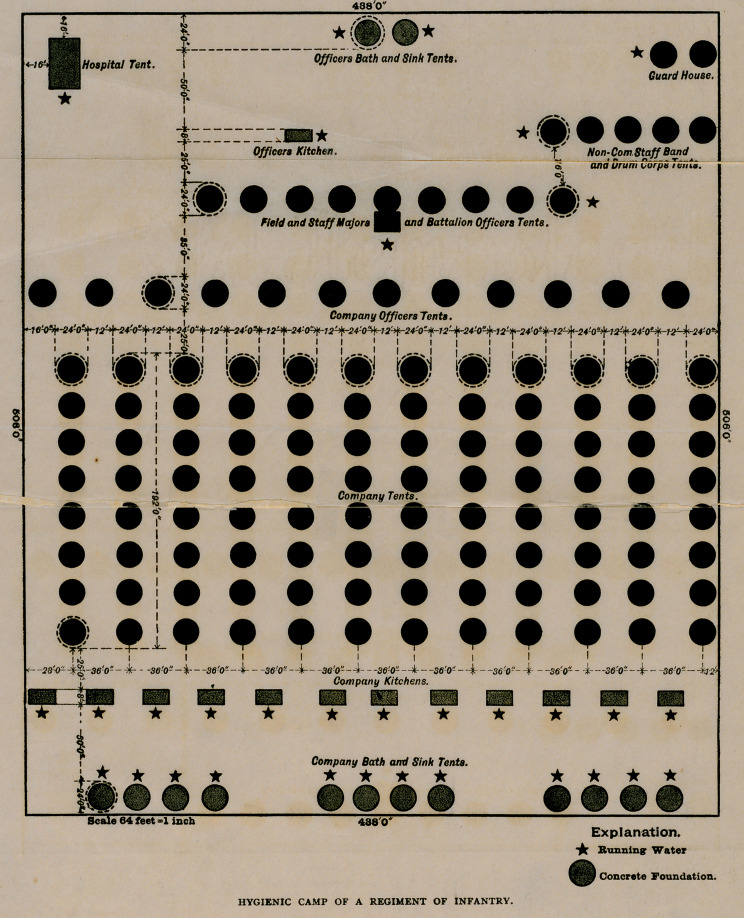# Camps of Instruction1Read at the regular meeting of the Buffalo Sanatory Club, December 14, 1898.

**Published:** 1899-02

**Authors:** Peter C. Harris

**Affiliations:** First Lieutenant and Quartermaster 13th Infantry, U. S. Army


### CAMPS OF INSTRUCTION.[1]

By PETER C. HARRIS,
First Lieutenant and Quartermaster 13th Infantry, U. S. Army.

THE object of the meeting has my most hearty approval, and to be selected to discuss the subject with men of such ability as those I see before me tonight, is an honor which I appreciate most highly. I regret, however, that the demands upon my time have been so great that I am compelled to present a hastily prepared paper, covering only the most important points of my subject.

The necessity for camps of instruction has been made so apparent in our late war with Spain that any remarks I might make would seem almost superfluous. They are required for the regular army as well as for the national guard or militia of the several states. They are even more beneficial to officers than to enlisted men.

For the regular army, camps of instruction are intended to supplement instruction received at posts. It is presumed that officers and men are thoroughly drilled in close order and extended order, at least to include the company and to some extent in outpost and advance and rear guard duty; therefore, for the regular army, such camps should resemble the autumn maneuvers of the European armies. On the other hand, the national guard or militia, except those from larger cities, must begin with the school of the soldier.

"Military drill is intended (1) to instruct the man in certain movements for his greater efficiency as a soldier acting with others and (2) to develop a certain power of physical endurance." A young recruit cannot keep pace with a full-grown and completely trained man in the ranks, mainly because his heart and blood-vessels are not fully developed nor specially trained." (Military Hygiene, Woodhull.) The time required to train a soldier is difficult to determine. Until within the past few years recruits for the regular army were sent to recruiting depots, where they were drilled for four months by specially selected officers and noncommissioned officers before they were assigned to regiments or companies. After arrival at the posts of their companies, they were still drilled as recruits from two to four and sometimes six months, before they were placed in ranks with the old soldiers. They were not yet considered trained soldiers, but were sufficiently advanced to receive their instruction with the other men of the company. In 1894 these recruiting depots were closed and now recruits are sent at once to join their companies, but are drilled separately as recruits from four to eight months.

1 Read at the regular meeting of the Buffalo Sanatory Club, December 14, 1898.

We cannot, of course, hope to have the national guard devote so much time to the preliminary instruction of the soldier. But how much instruction should be given them ?

The militia of England can be called out from three to four weeks annually and the period can be extended to eight weeks. Recruits on joining the volunteers of England attend thirty drills, and afterward, as a minimum, they must attend nine drills annually. The annual training period of the Canadian militia extends over twelve days.

No continental nation has a military force corresponding exactly to our national guard, but certainly the latter should receive at least as much training and instruction as men who are simply intended to fill vacancies in the reserve of the regular army of these nations or to form part of the army of the second line. The ersatz reserve of the continental armies receive from two to four months' training. The Landwehr, of Austria, " consists mostly of men who have completed ten years' service in the active army and in reserve; ten years' service in the ersatz reserve; and such men as, escaping the annual contingent, are at once assigned to it for the twelve years, during which they are liable to military service. . . . . .

" The men of the landwehr are required to attend one muster a year. The recruits who annually join receive eight weeks' training at battalion headquarters before being sent on furlough. There is also a company drill of a fortnight each year after the harvest, and every two years there is battalion instruction for three weeks, during which time the battalions must take part in the army maneuvers." (The Armies of Asia and Europe.) I have explained more fully the instruction of the landwehr of Austria because, in my opinion, this would be an admirable system to adopt for our national guard.

The company drill and a part of the recruit drill might be distributed throughout the year and in a city might be at night in the armory. The importance of thorough instruction of the recruit cannot be overestimated. Recruits of the national guard should during the summer or fall be sent to camps of instruction for at least four weeks and those from the country and smaller towns, where instruction is not continued throughout the year, should be given eight weeks' instruction in these camps. One half of the national guard of the state should be called out each year for three weeks' instruction. In states which have only two or three regiments it might be best to call out the entire guard every two years. But, it may be said, no state would be willing to expend so much on its soldiers. Congress should meet this objection by increasing the annual appropriation for the militia from four hundred thousand dollars to at least ten or twenty times that amount. The United States, as a whole, is or should be far more interested in the instruction of the national guardsman than any state can be.

The sites for camps of instruction should be selected by a board composed of a line officer, a surgeon and an officer of the quartermaster's department. I shall not presume to discuss the sanitary requirements of the site, but will pass at once to transportation and supplies.

The order for the concentration of the regular army at Tampa and other points in April last, directed that rations for one month, a limited amount of subsistence stores for sale to officers and enlisted men, and all wagon transportation be taken from the different military posts. As companies cannot very well take care of such a quantity of rations in the field, this order virtually required each regimental commissary to keep a store-house and make issues as well as sales of subsistence stores. This system was soon abandoned, but it has this to say in its favor—namely, that while at Tampa there was not a day that the men of the 13th Infantry failed to secure full rations, including fresh beef and vegetables. After leaving Tampa we did not fare so well.

At Montauk Point there was one commissary depot from which rations were issued every five days. For camps of instruction this would certainly be the best method. There should also be a temporary depot of quartermaster supplies at each camp.

As previously stated, all wagon transportation was taken from the different military posts to Tampa. This gave some regiments twenty or more wagons, but in most cases the regiments had less than half that number and the 13th Infantry had just two four-horse wagons. After a while our horses were taken away and mules substituted, but there was never a time at Tampa, or in fact during the war, when the regiment had more than two wagons. In Cuba we had no transportation whatever with the regiment. At Tampa we were informed that each regiment of infantry would be given twenty-five army or six mule wagons, the allowance fixed by general orders, but instead of issuing more all regiments were limited to two wagons, and brigade wagon trains were formed. This gave each brigade a wagon train of about thirty wagons and each regiment two wagons. There were also six or seven trains of pack mules at Tampa, but they were not sufficiently trained to be of any service until about the time we left for Cuba.

W1 BU424G
after p. 494

HYGIENIC CAMP OF A REGIMENT OF INFANTRY.

At Montauk Point each regiment of infantry, in our division at least, had two four-mule wagons. All other transportation, about one hundred and thirty wagons, was under the immediate order of the chief quartermaster. I have not included the transportation with the cavalry and artillery. Every regimental quartermaster and commissary condemned the system used at Tampa and Montauk Point. The transportation should be distributed to the regiments.

From the camps of instruction the regiments should make practice marches under conditions as near as practicable to what would be expected in actual warfare, and certainly they should have the transportation fixed by general orders as the minimum allowance for a regiment in the field. The general order referred to, which was issued May 25, 1898, also fixes the allowance of transportation for the headquarters of a brigade, division and army corps.

I will close with a few remarks upon the plan of the survey of a camp. The plan I have here is taken from the infantry drill regulation. (See illustration.) The distance and intervals are mine. None are given in the drill regulation, as they vary so much with the nature of the ground and the strength of the command. The tents are placed farther apart than is usually found in practice, but for camps that are to be occupied more than a week this is necessary. Once each week the tents should be moved to a new site and the old site scraped and exposed to the sun. To enable this to be done the space between the tents is made equal to the width of the tent. The tents of each company are usually arranged in two lines facing each other, but they may be arranged in one line, all facing in the same direction. The plan I have is made on a basis of three men to a common tent or ten to a conical wall tent. The minimum allowance of tentage is six men to a common tent and twenty to a conical wall.

FORT PORTER, BUFFALO, N. Y.

## Figures and Tables

**Figure f1:**